# Slow-velocity eccentric-only resistance training improves symptoms of type 2 diabetic mellitus patients by regulating plasma MMP-2 and -9

**DOI:** 10.1097/MD.0000000000038855

**Published:** 2024-07-19

**Authors:** Zhao Qian, Liu Ping, Xu Dongming, Zhang Xuelin

**Affiliations:** a College of Physical Education, Qufu Normal University, Qufu, China.

**Keywords:** eccentric-only resistance training, extracellular matrix, MMP-2 and -9, skeletal muscle, type 2 diabetic mellitus

## Abstract

**Objective:**

This study investigated the intervention effect of slow-velocity eccentric-only resistance training on type 2 diabetic mellitus (T2DM) patients based on the role of matrix metalloproteinase-2 and -9 (MMP-2 and -9) in regulating extracellular matrix homeostasis.

**Methods:**

50 T2DM patients were randomly divided into the slow-velocity eccentric-only resistance training group (E) and control group (C). The E group performed eccentric-only resistance training 3 times a week, every other day for 10 weeks, while the C group did not. Blood samples were collected before and after training, and subjects were tested for changes in clinical parameters, insulin resistance indices [fasting insulin, homeostatic model assessment insulin resistance (HOMA-IR)], MMP-2 and -9, and hydroxyproline, and muscle strength (12-RM), respectively.

**Results:**

After 10 weeks of training, the E group showed significant decreases in fasting glucose (*P* < .05), insulin (*P* < .05), insulin resistance indices (*P* < .05), hemoglobin A1c (HbA1c) (*P* < .01), triglycerides (*P* = .06) and MMP-2 (*P* < .05), while total cholesterol (*P* < .05), MMP-9 (*P* < .05), hydroxyproline (*P* < .01), Creatine Kinase (CK) (*P* < .05), and muscle strength (*P* < .001) significantly increased. There were no significant changes in the count of neutrophil, lymphocyte and platelet, neutrophil-to-lymphocyte ratio (NLR), platelet-to-lymphocyte ratio (PLR), high-density lipoprotein cholesterol (HDL-c), and low-density lipoprotein cholesterol (LDL-c). Compared with the C group, the E group showed a trend of a significant decrease in triglyceride (*P* < .05), lymphocyte count (*P* < .05), fasting glucose (*P* = .07), and plasma MMP-2 (*P* < .05), while MMP-9 (*P* < .05), hydroxyproline (*P* < .001), and muscle strength (*P* < .01) significantly increased. However, no significant changes were observed in insulin and insulin resistance indices, HbA1c, total cholesterol, HDL-c, LDL-c, CK, and other inflammatory indicators.

**Conclusions:**

Slow-velocity eccentric-only resistance training was beneficial for T2DM, but the potential role of MMP-2 and -9 in regulating extracellular matrix homeostasis is very different in T2DM patients.

## 1. Introduction

Type 2 diabetic mellitus (T2DM) is one of the most common chronic diseases, and to date, the pathogenesis of T2DM is still not well understood,^[1]^ but many scholars believe that skeletal muscle insulin resistance is the cause of the development of T2DM,^[2–6]^ and the main reason is related to the deposition of collagen fibers in the extracellular matrix (ECM)^[7]^ of skeletal muscle due to the inflammatory response caused by a high-fat diet.^[3,8,9]^ Members of the matrix metalloproteinases (MMPs) family, especially MMP-2 and -9, play a very important role in skeletal muscle ECM homeostasis^[10,11]^ due to their ability to degrade skeletal muscle ECM collagen fibrillar proteins.^[12]^ Many findings believe that the correlation between insulin resistance in skeletal muscle and collagen fiber deposition in ECM is attributable to the dysregulated expression of MMPs. Exercise is a universally accepted component of the nonpharmacologic treatment for T2DM. Studies have shown that resistance training maintains appropriate expression of skeletal muscle MMP-2 and -9 compared to aerobic endurance training,^[13,14]^ which is more effective in improving T2DM.^[15]^ Another study showed that eccentric contraction training increased skeletal muscle MMPs expression more compared to concentric contraction training,^[16]^ which may promote skeletal muscle ECM remodeling^[17]^ and improve skeletal muscle insulin sensitivity.^[12]^ Currently, although a large number of studies have shown that resistance training improves T2DM, few studies have been reported on the effects of eccentric training on people with T2DM^[18]^ and its mechanisms. In this study, we investigated the feasibility and mechanism of slow-velocity eccentric-only resistance training on T2DM based on the results of insulin-resistant ECM collagen deposition in skeletal muscle with T2DM and the remodeling effect of eccentric training on skeletal muscle based on the role of MMP-2 and -9 in regulating extracellular matrix homeostasis. (Note: slow-velocity eccentric-only resistance exercise was used in this study, because fast-velocity eccentric-only exercise would cause an increase in skeletal muscle ECM thickness^[19,20]^ and muscle atrophy^[21,22]^ compared to slow-velocity eccentric-only exercise, which may exacerbate the degree of insulin resistance).

## 2. Methods

### 2.1. Patients

**Figure 1.**
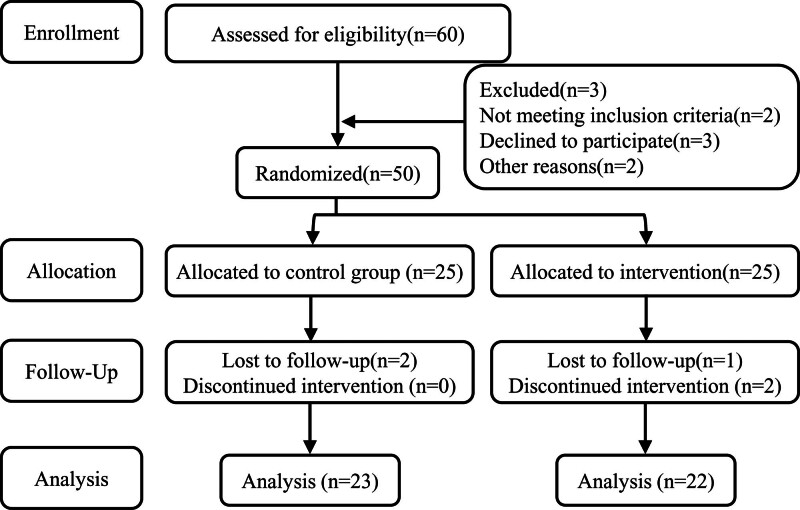
Flow diagram.

T2DM patients (oral glucose tolerance test of ≥ 11.1 mmol/L or fasting blood glucose of ≥ 7 mmol/L) were recruited.^[22]^ All recruited T2DM subjects need to be tested for a physical checkup. By screening, 50 T2DM patients (32 men, 18 women, 51–66 years old) were selected as the study subjects, and were randomly divided into the slow-velocity eccentric-only resistance training group (E) and control group (C) using a random number generator. Due to the withdrawal, finally, there were 23 patients in the C group and 22 patients in the E group. All participating in this experimental study signed an informed consent form. The Ethics Committee of Qufu Normal University approved and supervised the study. The flow chart was shown in Figure 1.

Data of T2DM patients were collected at Qufu People’s Hospital. A wide range of information was gathered, including personal details, disease progression, laboratory reports, administered treatments, and official diagnoses. All of this data was meticulously recorded and stored within a secure and nonpublic Electronic Medical Records System. The collected data will be critical to promote a deeper understanding of T2DM and support future study aimed at improving T2DM patient care.

The study excluded T2DM subjects who had the following conditions: severe cardiovascular disease (including unstable angina, severe arrhythmias, and transient ischemic attacks), central or peripheral nervous system disease, severe orthopedic disease (such as a rheumatological condition that limited mobility or caused knee flexion impairment of less than 90°), diabetic retinopathy, myopathy, severe renal disease, uncontrolled hypertension (blood pressure ≥ 140/90 mm Hg), excessive hemoglobin A1c (HbA1c > 10%), and those who required insulin injections.

### 2.2. Training protocols

#### 2.2.1. Eccentric-only resistance training models

The following is the specific implementation, using the resistance equipment training method to design 5 exercise models.

##### 2.2.1.1. Eccentric-only exercise of seated leg curl

**Figure 2.**
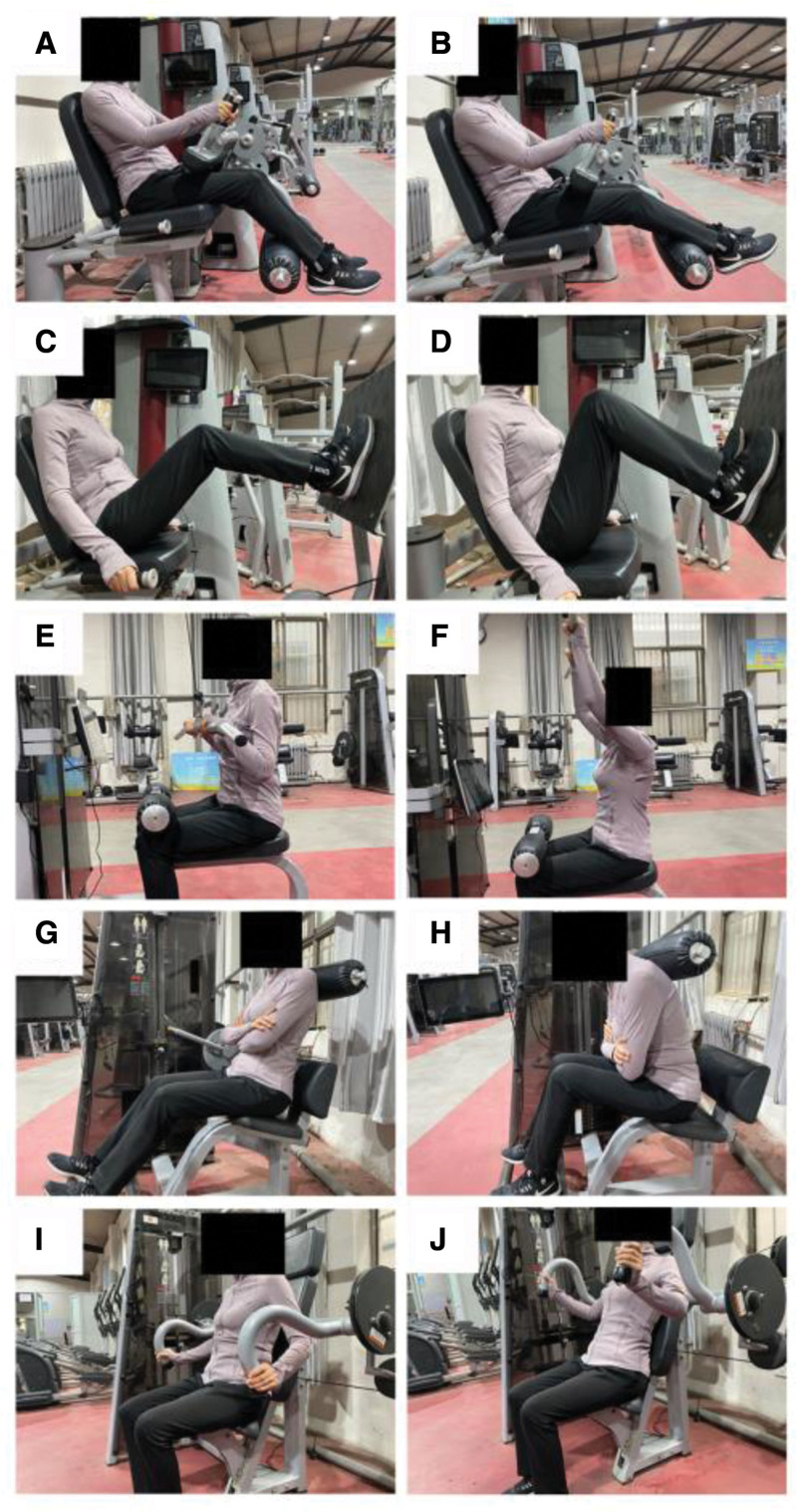
Eccentric resistance training models.

The subjects sat on the sitting leg curl training machine, held the handles for support, the back pressed against the back pad, and the posterior side of the lower calves pressed over the padded lever. Researchers completed the downward press of the padded lever (Fig. 2A), and the participants only needed to complete the reset movement against the padded lever upwards within the specified time (Fig. 2B).

##### 2.2.1.2. Eccentric-only exercise of seated leg press

The subjects assumed a seated position on the leg press training machine, with their backs against the back pad and holding onto the handles for support. They bent their legs to push the pedals before their body while maintaining a slight knee bend at the top. The researchers performed the pedal-pushing action (Fig. 2C), and the participants were only required to execute the reset movement against the padded lever within the specified time (Fig. 2D).

##### 2.2.1.3. Eccentric-only exercise of seated pull-down

The subjects positioned themselves on the high-stretch training machine, grasping the handles with both hands. The researchers performed the downward press of the padded lever (Fig. 2E). Simultaneously, the participants were responsible for executing the reset movement by pushing the padded lever upwards within the designated time frame (Fig. 2F).

##### 2.2.1.4. Eccentric-only exercise of seated back extension

The subjects were seated on the back extension machine, positioning their back against the padded lever. The researchers actively initiated the downward press of the padded lever (Fig. 2G) while the participants transitioned from the seated position to the semi-supine position using the rotating bearing. Subsequently, the participants were required to actively perform the reset movement by pushing the padded lever upwards within the designated time (Fig. 2H).

##### 2.2.1.5. Eccentric-only exercise of seated arm flexion and extension

The subjects positioned themselves on the sitting triceps training machine and grasped the handles with both hands. The researchers performed the downward press of the handles (Fig. 2I), while the participants were responsible for executing the upward reset movement against the handles within the specified time (Fig. 2J).

#### 2.2.2. Eccentric-only resistance training program

Subjects in the E group perform 2 weeks pre-adaptation + 8 weeks of formal training intervention while maintaining routine daily dietary and drug treatment procedures. The specific implementation is as follows.

##### 2.2.2.1. Pre-adaptation training program

Subjects underwent a 2-week pre-adaptation training phase before the formal training commenced. During this phase, the subjects selectively increased the resistance load and training duration. The perceived exertion during this pre-adaptation training was within the “very light” or “light” range on the Borg Rating of Perceived Exertion Scale (BRPES).^[23]^ The training frequency was 3 times per week, with one day of rest between sessions. During the first week, the individual completed one set of exercises lasting 15 to 30 minutes. The exercise load varied between 20% and 40% of their One Repetition Maximum. From the second week onwards, the training lasts for two sets ranging from 30 to 60 minutes each, with the exercise load between 30% and 50% of their One Repetition Maximum.

*Precautions* The physiological parameters of the participants, namely their blood glucose levels, resting blood pressure and heart rate, were meticulously recorded and documented in their corresponding exercise log sheet. A pre-exercise blood pressure < 140/90 mm Hg was required for the subjects to begin the exercise. If the blood glucose level was < 5.5 mmol/L, they needed to supplement with appropriate carbohydrates or exercise for 20 to 30 min and reassess blood glucose levels to ensure they were not dropping. If the pre-exercise blood glucose level was > 16.7 mmol/L, subjects began an exercise and were reassessed in 20 to 30 minutes to ensure the blood glucose level was not increasing. If the blood glucose level increases, the exercise will stop. For pre-exercise training, subjects performed 5-min warm-ups with a light exertion level on BRPES. Emphasize various adverse reactions that may occur during training and remind subjects to give positive feedback if they feel uncomfortable.

##### 2.2.2.2. Formal training program

After completing 2 weeks of pre-adaptation training, we measured the subject’s 12-RM load for the eccentric-only contraction in 5 exercise modes: seated leg curl, seated leg press, seated pull-down, seated back extension, and seated arm flexion and extension. The intervention involved an 8-week slow-velocity eccentric-only exercise training program using the 12-RM load. Each training session consisted of a 10-minute warm-up and 2 sets of eccentric-only resistance training lasting 45 minutes. A 5-minute relaxation activity concluded the session. Subjects scheduled training sessions on Mondays, Wednesdays, and Fridays and took breaks on Saturdays and Sundays.

During the training sessions using the 12-RM load, the duration of eccentric-only contraction progressively increased as follows: 10 seconds for the first and second week, 12 seconds for the third and fourth weeks, 15 seconds for the fifth and sixth week, and 17 seconds for the seventh and eighth week. Subjects took breaks of 3 to 5 minutes between sets and a 2 to 3 minutes rest between each exercise mode.

*Precautions* During eccentric training, the BRPES presents a challenging level of exertion. Before each eccentric training session, a Visual Analogue Scale (VAS) was utilized to monitor the intensity of muscle pain. If there was an increase in muscle pain, we took proactive measures to suspend the eccentric training. In addition, the healthcare providers frequently monitored the participants to promptly identify any risks associated with low blood sugar or sudden diabetic problems. This monitoring was thorough and continuous. It was imperative to closely supervise the subjects throughout the training sessions. We emphasized the importance of prompt responses from the subjects if they experienced discomfort or adverse symptoms. If someone shows signs of hypoglycemia, immediate treatment involves the administration of sugar and carbohydrates to alleviate the symptoms.

### 2.3. Sample collection

A senior laboratory technician collected a fasting blood sample of 15 mL before the pre-adaptation training and the following morning of the eighth weekend of the formal 8 weeks training, from 8:00 to 9:00 am. A portion of the blood sample, specifically 8 ml, was utilized for testing routine blood parameters such as leukocyte count, neutrophil count, lymphocyte count, platelet count, fasting glucose, triglycerides, total cholesterol, high-density lipoprotein cholesterol (HDL-c), and low-density lipoprotein cholesterol (LDL-c), insulin, and HbA1c.

Another portion of the blood sample, 7 mL, was used to detect MMP-2 and -9 levels and hydroxyproline concentration. The blood sample was centrifugated at 3000 r/min for 10 minutes to separate the serum and then divided into several aliquots. A portion of the sample was measured immediately to determine the Creatine Kinase (CK) levels, and the remaining serum samples were kept in a refrigerator at −20°C for future testing.

Furthermore, the subject’s height, weight, and blood pressure were measured at the same exercise time before the pre-adaptation training and on the day following the eighth weekend of the formal 8-week training.

### 2.4. Indicator testing and methods

#### 2.4.1. Measurement of body morphometric indicators

Height (m) and weight (kg) were measured by using a height and weight measuring device. The body mass index was calculated based on Body Mass Index, BMI = [weight (kg)]/[height (m^2^)].

#### 2.4.2. Eccentric-only resistance 12-RM load test methods

Referring to the protocol of Beam et al^[24]^ with slight modifications, the specific steps were: 5 to 10 minutes aerobic warm-up, light stretching, and 3 repetitions of submaximal strength exercises; 4 trial lifts were used to determine a 12-RM load with 3 to 5 minutes rest between trials; the appropriate initial lifting weight was selected; the maximum number of repetitions for the final 12 times was 12-RM, and the load was 12-RM load at this time. Note: Only 4 trial lifts can be performed. Otherwise, it will be performed on the next day.

#### 2.4.3. Training intensity monitoring indicator measurements

The BRPES was used to monitor the degree of subjective exertion during exercise. The sensory scale was divided into 9 levels from 6 to 20, and the corresponding value of subjective exertion of the subjects in this study was between 11 and 13. Note: Researchers should avoid using introductory language to force subjects. The VAS was used to measure muscle damage indirectly. Muscle pain was determined (in centimeters) by the use of a 10 cm VAS anchored between 0 cm (“no pain”) and 10 cm (“worst possible pain”). Subjects marked their leg muscle pain under the guidance of researchers before and after each training session.

#### 2.4.4. Measurement of blood indicators

The double-antibody one-step sandwich enzyme-linked immunosorbent assay (ELISA) method was used to test the contents of MMP-2 and -9, CK, HbA1c and hydroxyproline (Duma Biotechnology, Shanghai, China). The ELISA test kit instructions were strictly followed during the experiment. The specific steps were as follows: the frozen serum was taken, thawed at room temperature, and the specimen, standard, and horseradish peroxidase (HRP)-labeled detection antibodies were added to the pre-covered microtiter wells with antibodies of MMP-2 and -9, CK, HbA1c and hydroxyproline in turn, after incubation and thorough washing. Color development was performed with the substrate TMB, which was converted to blue by peroxidase catalysis and to a final yellow color by acid. The shade of color was positively correlated with the HbA1c in the sample. The absorbance was measured at 450 nm wavelength using a microplate reader, and the sample concentration was calculated.

##### 2.4.4.1. Insulin resistance test method

The degree of insulin resistance was evaluated using the HOMA-IR, HOMA-IR = [fasting insulin (mIU/L) × fasting glucose (mmol/L)]/22.5.

### 2.5. Statistical analysis

All results were presented as means ± SD. The Mann–Whitney *U* test tested the clinical parameters with two independent samples of the non-parametric test. Comparisons within groups were performed using a paired-sample *t* test. Comparisons between groups were performed using Student’s independent *t* test. A two-tailed *P* < .05 was considered statistically significant. The statistical analyses were carried out using SPSS 19.0 (IBM, Armonk). Images were created and processed using Origin Pro 9.0 and Photoshop CS6.

## 3. Results

### 3.1. Clinical, blood glucose and lipid parameters

The general clinical parameters (BMI, blood pressure) of both the E group and C group did not show significant changes before training (Table 1). After 10 weeks of training (Table 2), there were significant trends of decreasing fasting glucose (*P* < .05), insulin (*P* < .05), insulin resistance indices (*P* < .05), HbA1c (*P* < .01), and triglyceride (*P* = .064), while total cholesterol (*P* < .05) significantly increased in the E group. LDL-c and HDL-c did not show significant changes in the E group and the C group. The E group showed a noticeable trend of reduced fasting glucose (*P* = .07) and triglyceride (*P* < .05) levels compared to the C group. In contrast, the E group found no significant decrease in insulin, insulin resistance indices, HbA1c, total cholesterol, LDL-c, and HDL-c. Regarding the percentage decrease in fasting blood glucose, the E group showed a higher percentage decrease than the C group. However, the percentage changes in total cholesterol, LDL-c, and HDL-c in the E group were contrary to conventional expectations. Total cholesterol and LDL-c increased by 17% and 6% in the E group and decreased by 3% and 6% in the C group, respectively. HDL-c decreased by 8% in the E and 5% in the C groups (Table 2). Based on these results, the effect of slow-velocity eccentric-only resistance training on lipid metabolism was paradoxical and not favorable. Eccentric exercise could change the ECM in skeletal muscle, potentially linking to this result. This transformation called for more lipids to fix damaged cells like myocytes and endothelial cells.

**Table 1:** Baseline characteristics of the type 2 diabetic mellitus patients.

	Exercise group (n = 22)	Control group (n = 23)	*P*
BMI (kg/m^2^)	24.76 ± 0.84	24.73 ± 0.87	.955
Systolic blood pressure (mm Hg)	142.10 ± 7.76	141.70 ± 8.53	.336
Diastolic blood pressure (mm Hg)	87.40 ± 5.55	88.67 ± 5.06	.694
Diabetes duration (yr)	6.38 ± 5.42	7.50 ± 4.85	－
Smoking (n)	2	4	－
Antihypertensive medications (n)	3	5	－
Oral antidiabetic drugs (n)	6	7	－
Sulfonylureas (n)	1	2	－
Metformin (n)	4	3	－
Other drugs (n)	1	2	－

3 subjects in C group and 4 subjects in E group did not take hypoglycemic drugs, respectively; －indicates that statistical analysis cannot be performed.

BMI = body mass index.

**Table 2:** Clinical and biochemical parameters of type 2 diabetic mellitus patients.

	Control group (n = 23)			%△	Exercise group (n = 22)			%△	*P* ^2^
	Baseline	10 weeks	*P* ^1^		Baseline	10 weeks	*P* ^1^		
BMI (kg/m^2^)	24.73 ± 0.64	24.27 ± 0.64	.103	2 ↓	24.76 ± 0.84	24.19 ± 1.22	.154	2 ↓	.233
Systolic blood pressure (mm Hg)	141.37 ± 8.53	143.67 ± 8.33	.749	2 ↑	142.10 ± 7.76	139.67 ± 9.84	.148	2 ↓	.889
Diastolic blood pressure (mm Hg)	88.67 ± 5.06	85.00 ± 6.03	.291	4 ↓	87.40 ± 5.55	77.20 ± 14.38	.088	12 ↓	.413
Fasting blood glucose (mmol/L)	9.47 ± 2.57	9.87 ± 2.23	.572	4 ↑	9.38 ± 2.49	8.42 ± 1.54	.016*	10 ↓	.070
Insulin (mIU/L)	11.45 ± 7.90	12.05 ± 8.71	.390	5 ↑	11.83 ± 6.21	8.76 ± 4.10	.028*	26↓	.343
Insulin resistance indices	4.85 ± 2.86	4.94 ± 2.05	.910	4 ↑	4.65 ± 2.05	3.28 ± 1.40	.016*	29 ↓	.272
Triglyceride (mmol/L)	2.77 ± 0.54	3.07 ± 1.69	.986	11 ↑	2.63 ± 0.40	2.32 ± 0.62	.064	12 ↓	.024*
Total cholesterol (mmol/L)	5.39 ± 1.00	5.25 ± 0.91	.153	3 ↓	5.21 ± 1.30	6.08 ± 0.93	.031*	17 ↑	.818
HDL-C (mmol/L)	1.65 ± 0.28	1.46 ± 0.18	.325	5 ↓	1.53 ± 0.91	1.41 ± 0.33	.992	8 ↓	.635
LDL-C (mmol/L)	3.37 ± 0.88	3.18 ± 1.05	.186	6 ↓	3.32 ± 1.26	3.53 ± 1.02	.301	6 ↑	.924
HbA1c (%)	7.93 ± 1.70	7.77 ± 1.76	.184	2 ↓	7.68 ± 1.43	7.05 ± 1.38	.006**	7 ↓	.265
leukocyte count (10^9^/L)	7.11 ± 1.20	7.80 ± 1.53	.155	10 ↑	7.25 ± 2.13	6.95 ± 1.42	.462	4 ↓	.759
Neutrophil count (10^9^/L)	3.98 ± 0.75	4.54 ± 1.15	.190	14 ↑	3.93 ± 1.21	3.52 ± 1.05	.158	10 ↓	.473
Lymphocyte count (10^9^/L)	2.73 ± 0.23	2.78 ± 0.10	.664	2 ↑	2.69 ± 0.78	2.54 ± 0.53	.767	6 ↓	.050*
Platelet count (10^9^/L)	221.00 ± 66.19	225.00 ± 81.96	.510	2 ↑	217.60 ± 64.93	232.80 ± 60.42	.240	7 ↑	.563
NLR (%)	1.45 ± 0.20	1.57 ± 0.46	.298	8 ↑	1.38 ± 0.28	1.51 ± 0.25	.073	9 ↑	.821
PLR (%)	89.21 ± 24.87	91.36 ± 34.36	.811	2 ↑	87.33 ± 9.59	95.26 ± 25.06	.131	9 ↑	.968

*P*^1^: changes of variables within-groups; *P*^2^: changes of variables between-groups; %△: the percentage of changes before and after variables.

BMI = body mass index, HbA1c = hemoglobin A1c, HDL-c = high-density lipoprotein cholesterol, LDL-c = low-density lipoprotein cholesterol, NLR = neutrophil-to-lymphocyte ratio, PLR = platelet-to-lymphocyte ratio.

* *P* < .05.

** *P* < .01.

### 3.2. Inflammation, MMP-2, MMP-9, hydroxyproline and CK parameters

**Figure 3.**
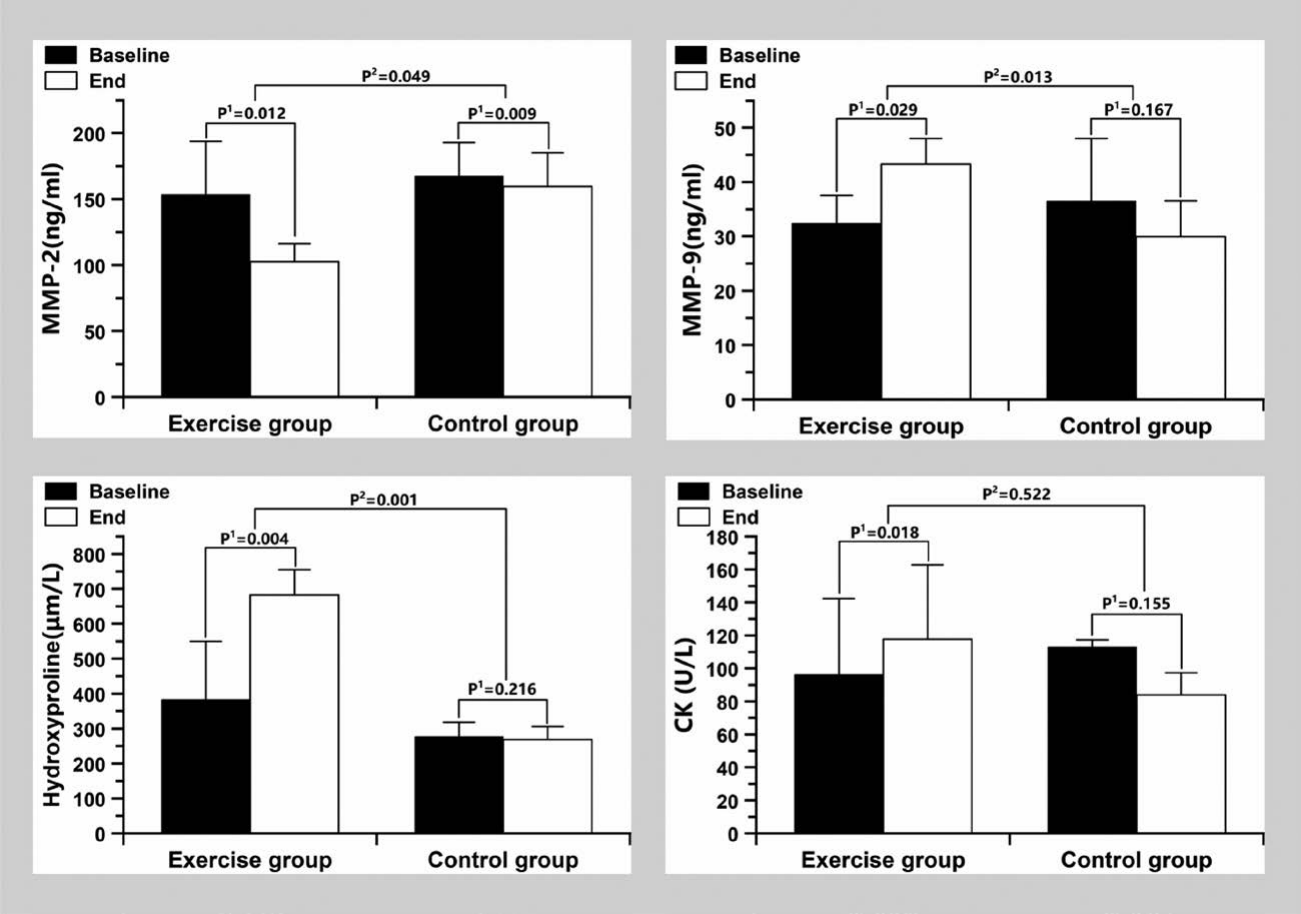
Plasma MMP-2, MMP-9, Hydroxyproline and CK levels in type 2 diabetic patients in training group and control group at baseline and at the end of the study. Data are expressed as means ± SD. *P*^1^: changes of variables within groups; *P*^2^: changes of variables between groups.

Compared with 10 weeks before, there were significant increasing trend was observed in plasma MMP-9 (*P* < .05), hydroxyproline (*P* < .01) and CK (*P* < .05), and MMP-2 (*P* < .05) significantly decreased in E group, while there were no significant changes in C group (Fig. 3). A significant decreasing trend was observed in lymphocyte count (*P* < .05) (Table 2) and MMP-2 (*P* < .05), while MMP-9 (*P* < .05) and hydroxyproline (*P* < .001) significantly increased in E group compared with the C group (Fig. 3). Compared with the C group, although there were no significant changes in the count of leukocyte, neutrophil and platelet, as well as neutrophil-to-lymphocyte ratio (NLR) and platelet-to-lymphocyte ratio (PLR) in the E group; the percentage change in the E group was much higher than in the C group in terms of the percentage change (increase or decrease), which showed two states. One state was a decrease, i.e., the leukocyte count and neutrophil count decreased by 4% and 10% in the E group, while increased by 10% and 14% in the C group, respectively. The other state was an increase, i.e., the platelet count, NLR, and PLR increased by 7%, 9%, and 9% in the E group, respectively, while increased by 4%, 8%, and 2% in the C group, respectively (Table 2). These results indicated that slow-velocity eccentric resistance training specifically improved blood inflammation in T2DM patients, but it needed to be more prominent. Interestingly, after 10 weeks of training, the levels of MMP-9 (*P* < .05) and hydroxyproline (*P* < .05) showed a significant increase, while MMP-2 (*P* < .05) exhibited a significant decrease in T2DM patients. Furthermore, the serum CK levels increased (*P* < .05) but remained within the normal range (Fig. 3). These findings suggest that slow-velocity eccentric-only resistance training induced skeletal muscle remodeling. In summary, the results demonstrated that slow-velocity eccentric-only resistance training had a limited impact on improving the inflammatory response in T2DM. However, it did significantly alter hydroxyproline, MMP-2 and-9 levels, indicating changes in ECM remodeling.

### 3.3. Muscle strength

After 10 weeks of training (Table 3), the 12-RM values of seated leg press (*P* < .001), seated back extension (*P* < .001), seated arm flexion and extension (*P* < .001), seated pull-down (*P* < .001) and seated leg curl (*P* < .01) were significantly increased in the E group; while the 12-RM value of seated leg curl (*P* < .05) was significantly increased in the C group. Compared with the C group, the 12-RM values of seated leg press (*P* < .01), seated back extension (*P* < .01), seated arm flexion and extension (*P* < .01), seated pull-down (*P* < .01) and seated leg curl (*P* < .05) were significantly increased in the E group (Table 3). According to the above results, slow-velocity eccentric-only resistance training substantially increased muscle strength in T2DM patients.

**Table 3:** Muscle strength (12-RM) of type 2 diabetic mellitus patients.

	Control group (n = 23)		*P* ^1^	Exercise group (n = 22)		*P* ^1^	*P* ^2^
	Baseline	10 weeks		Baseline	10 weeks		
Seated pull-Down	51.90 ± 9.46	54.50 ± 7.32	.073	52.40 ± 9.99	61.90 ± 10.38	.001***	.01**
Seated leg curl	67.80 ± 15.51	69.50 ± 14.57	.05*	66.50 ± 15.20	77.40 ± 16.15	.01**	.05*
Seated leg press	99.50 ± 13.15	103.20 ± 10.69	.402	100.70 ± 12.46	112.20 ± 9.54	.001***	.01**
Seated arm flexion and extension	62.80 ± 9.07	65.50 ± 8.95	.065	63.30 ± 8.97	73.40 ± 7.34	.001***	.01**
Seated back extension	81.90 ± 9.30	85.60 ± 9.11	.080	81.20 ± 10.41	93.50 ± 8.28	.001***	.01**

*P*^1^: changes of variables within-groups; *P*^2^: changes of variables between-groups.

12-RM = 12 Repetition Maximum.

* *P* < .05.

** *P* < .01.

*** *P* < .001.

## 4. Discussion

### 4.1. Intervention effect of slow-velocity eccentric-only resistance training on clinical symptoms of T2DM patients

This study observed significant reductions in fasting glucose, insulin, insulin resistance indices and HbA1c in T2DM patients who underwent slow-velocity eccentric-only resistance training (E group). These findings indicated that slow-velocity eccentric-only resistance training improved glucose-related indicators and enhanced insulin sensitivity in T2DM patients. These results aligned with previous research conducted by Ho et al.^[12]^ However, slow-velocity eccentric-only resistance training may have limited effectiveness in improving lipid-related indicators (lipid control level) in T2DM patients. This study suggested that the unique slow-velocity eccentric-only resistance training model may have less effect on lipid interventions.

Several studies have demonstrated that eccentric training can increase skeletal muscle capillary density. For instance, Rodriguez-Miguelez^[25]^ found that Wistar rats experienced a rise in capillary density in their vastus lateralis muscle after 8 weeks of eccentric endurance training of an intermittent downhill (−16°, 90 min) protocol. Other studies have also shown that a combination of resistance training (including eccentric exercise) and aerobic training could effectively enhance the vascular structure of skeletal muscle in T2DM patients. This improvement in small vessel function can ultimately lead to better control of blood glucose levels.^[26,27]^ Our study examined the effects of slow-velocity eccentric-only resistance training on skeletal muscle vascular endothelium. We observed a potential release of extracellular cholesterol into the bloodstream due to this training approach. This phenomenon may explain the abnormal changes in total cholesterol, LDL-c, and HDL-c levels.

In conclusion, slow-velocity eccentric-only resistance training has positively affected glucose-related indicators and insulin sensitivity in T2DM patients. However, the impact on lipid-related indicators may vary depending on the specific eccentric training model utilized, potentially leading to abnormal changes in cholesterol levels.

### 4.2. Mechanism analysis of slow-velocity eccentric-only resistance training in improving symptoms of T2DM

#### 4.2.1. Effect of slow-velocity eccentric-only resistance training on inflammatory indicators in T2DM patients

The above study has shown that slow-velocity eccentric-only resistance training can improve the clinical symptoms of T2DM patients, especially in blood glucose control, with significant effects. According to recent studies, chronic inflammation played a key role in the pathogenesis of diabetes and its complications.^[28]^ Therefore, this study aimed to delve deeper into the impact of eccentric resistance training on inflammatory markers among T2DM patients and elucidate the intervention’s efficacy mechanism. Our findings revealed that slow-velocity eccentric-only resistance training did not significantly improve inflammatory markers among T2DM patients. This lack of efficacy may be attributed to the potential for excessive eccentric training to induce skeletal muscle microdamage, subsequently triggering an inflammatory response within the body.^[5]^ The reason for the poor effect of eccentric resistance group training on the inflammatory response^[29]^ may be related to the neutralization of the exercise effect by the inflammatory response caused by eccentric training. However, although the CK value of the E group increased, it was within the normal range, which did not support the above idea. El-Kader^[30]^ provided different intervention mechanisms for aerobic endurance and resistance training to improve the symptoms of T2DM. T2DM subjects performed aerobic endurance training, which includes 60-80% of maximal heart rate endurance training on a running platform and resistance training of 3 × 8 to 12-RM for 12 weeks at 40 minutes each. It was found that HOMA-IR, HbA1c and inflammatory response indicators [TNF-α, interleukin-6 (IL-6)] were substantially reduced in both groups. However, the aerobic endurance training group had more effect on regulating insulin sensitivity, adipocytokines and inflammatory cytokines, and the resistance training group was more effective in increasing muscle circumference. Given the findings of El-Kader,^[30]^ resistance training was more effective in increasing muscle circumference, the study in this paper was confirmed, and it was found that skeletal muscle strength was a significant increase in the E group compared with the C group. The mechanism of the intervention effect of slow-velocity eccentric-only resistance training was closely related to the improvement of skeletal muscle mass. At the same time, it was less associated with the inflammatory response.

#### 4.2.2. Effect of slow-velocity eccentric-only resistance training on MMP-2 and -9 and hydroxyproline concentrations in T2DM patients

To some extent, the intervention effect mechanism of the slow-velocity eccentric-only resistance training was strongly associated with a significant downregulation of lymphocyte count in this study. Thus, the slow-velocity eccentric-only training intervention effect mechanism was still related to regulating the inflammatory response. Based on the latest research trend,^[31,32]^ the pathogenesis of T2DM was associated with the collagen fiber deposition of ECM by chronic inflammation-induced in skeletal muscle. Williams et al^[33]^ elaborated the causes, suggesting that the deposition of collagen fibers in the ECM not only increased the diffusion distance of glucose and insulin, but also impaired the vascular growth and function of skeletal muscle, which causes skeletal muscle vascular dysfunction and subsequent reduction in capillary density, inducing skeletal muscle insulin resistance and the development of T2DM.^[34,35]^ Numerous studies have demonstrated that MMP-2 and -9 play an important role in skeletal muscle ECM homeostasis due to their ability to degrade collagen fibronectin, the main component of skeletal muscle ECM, and most of the other proteins.^[13,36]^ Dysregulation of MMPs expression induced skeletal muscle insulin resistance and T2DM.^[36,37]^ However, after 8 weeks of rowing ergometer training (every other day, 30 min, 65–70% VO_2_ max) in T2DM patients, MMP-2 increased in vastus lateralis muscle.^[38]^ It implied that the mechanism of the slow-velocity eccentric training intervention effect was related to the regulation of MMP-2 and -9 activity, which was supported by this study. MMP-2 was significantly decreased, while MMP-9 and hydroxyproline were significantly increased in the E group. These results suggested that the mechanism of eccentric resistance training intervention effect in T2DM was related to regulating MMP-2 and -9 activity.

Recent studies have further corroborated this notion, demonstrating significant increases in MMP-2 and -9 concentrations among T2DM patients,^[39]^ and the dysregulated expression of MMPs has been seen as a cause of obesity and diabetes.^[40]^ Hyperglycemia and dyslipidemia in T2DM patients can contribute to vascular endothelial dysfunction, leading to increased expression of endothelial cells and macrophages derived from monocytes.^[41]^ Hyperglycemia and dyslipidemia in T2DM patients were generally believed to be the result of chronic inflammation caused by long-term immune system imbalance,^[42,43]^ in which macrophages are the main inflammatory cells.^[44]^ Early researchers usually attributed the inflammatory response to the imbalance of the natural immune system.^[45]^ However, recent studies have shown that the adaptive immune system, especially T-lymphocytes, played a vital role in the pathogenesis of T2DM and was related to activating macrophages.^[46–48]^

Our study found that slow-velocity eccentric-only resistance training led to a significant decrease in lymphocyte count. This type of resistance training may mitigate the chronic inflammatory response in T2DM patients by reducing lymphocyte count in the bloodstream, particularly macrophage activity, resulting in decreased plasma MMP-2 levels. Several studies have offered preliminary support for this hypothesis.^[49,50]^ For instance, research on elderly obese women revealed that acute eccentric resistance exercise (7 sets of 10 repetitions at knee extension maximal voluntary contraction) reduced plasma MMP-2 and -9 levels, along with downregulation of IL-6.^[50]^ However, the view of reduced lymphocyte count cannot explain the findings of increased plasma MMP-9. Recent studies have also provided new insights into the upregulation of MMP-9.^[51]^ Study observed that MMP-2 was distributed in different regions within myocytes, including the Z-line, nuclear membrane, and mitochondrial sites, whereas MMP-9 exclusively localized in the ECM.^[52]^ It was hypothesized that MMP-2 could be translocated outside the myocyte only when the myocyte was damaged during exercise training, but long-term eccentric training led to skeletal muscle adaptation,^[16,53]^ resulting in a decrease in the amount of MMP-2 translocated outside myocyte, which in turn may reduce the concentration of MMP-2 in the blood. This explained at another level the decrease in MMP-2 in the blood after eccentric resistance training. However, for MMP-9, given the findings of many scholars that eccentric training can cause skeletal muscle vascular remodeling,^[54,55]^ and because the skeletal muscle ECM was closely linked to the vascular endothelium, and MMP-9 was located in the skeletal muscle ECM.^[34,35]^ The hypothesis posited that the eccentric-only resistance training model at very slow velocity could induce enhanced remodeling of the skeletal muscle ECM, resulting in increased release of MMP-9 into the bloodstream. The significant increase in hydroxyproline levels strongly supported this observation pre- and post-training and between groups. Previous research has demonstrated the beneficial effects of slow-velocity eccentric-only training on T2DM patients, including reductions in inflammatory cytokines, and then the regulation of MMPs activity to maintain skeletal muscle ECM homeostasis.

## 5. Conclusion

Slow-velocity eccentric-only resistance training exhibited significant reductions in fasting glucose, insulin, insulin resistance indices, HbA1c, triglyceride and MMP-2, while demonstrating significant increases in total cholesterol, MMP-9, hydroxyproline, CK and muscle strength in T2DM patients. However, there were no notable effects on LDL-c, HDL-c, counts of neutrophil, lymphocyte and platelet, NLR and PLR. These findings suggested that slow-velocity eccentric-only resistance training actively modulated the process of remodeling skeletal muscle ECM, leading to improvements in T2DM symptoms. Notably, MMP-2 and -9 also played distinct roles in regulating ECM homeostasis.

## Acknowledgments

The authors would like to thank all study participants. We acknowledged the staff members who participated in health checkups and data entry.

## Author contributions

**Conceptualization:** Zhao Qian, Liu Ping, Zhang Xuelin, Xu Dongming.

**Data curation:** Zhao Qian, Xu Dongming.

**Formal analysis:** Zhao Qian, Zhang Xuelin, Xu Dongming.

**Funding acquisition:** Zhang Xuelin.

**Investigation:** Zhao Qian, Liu Ping, Zhang Xuelin, Xu Dongming.

**Methodology:** Zhao Qian, Liu Ping, Zhang Xuelin, Xu Dongming.

**Project administration:** Zhao Qian, Zhang Xuelin.

**Resources:** Zhang Xuelin.

**Software:** Zhao Qian.

**Supervision:** Zhao Qian, Zhang Xuelin.

**Validation:** Zhao Qian, Liu Ping, Zhang Xuelin, Xu Dongming.

**Visualization:** Zhao Qian, Liu Ping, Zhang Xuelin, Xu Dongming.

**Writing – original draft:** Zhao Qian, Liu ping, Zhang Xuelin, Xu Dongming.

**Writing – review & editing:** Zhao Qian, Liu Ping, Zhang Xuelin, Xu Dongming.
